# *Leuconostoc mesenteroides* subsp. strain NTM048 ameliorated nasal symptoms in patients with Japan cedar pollinosis: Randomized, double-blind, and placebo-controlled trial

**DOI:** 10.1097/MD.0000000000035343

**Published:** 2023-11-10

**Authors:** Yuta Yamamoto, Gen Sugita, Masanobu Hiraoka, Saori Takagi, Naoko Yamagishi, Shuji Kawashima, Kensuke Tanioka, Toshio Nishi, Sachi Yamamoto, Chiemi Kakutani, Agi Yanase, Yoshimitsu Kanai, Seiya Kato, Muneki Hotomi

**Affiliations:** a Department of Anatomy and Cell Biology, Wakayama Medical University, Wakayama, Japan; b Department of Otorhinolaryngology-Head and Neck Surgery, Wakayama Medical University, Wakayama, Japan; c Department of Emergency and Intensive Care Medicine, Wakayama Medical University, Wakayama, Japan; d Clinical Study Support Center, Wakayama Medical University Hospital, Wakayama, Japan; e Department of Nursing, Wakayama Medical University Hospital, Wakayama, Japan.

**Keywords:** allergic rhinitis, *Leuconostoc mesenteroides* subsp. strain NTM048, POMS2, psychological status, s-IgA

## Abstract

**Background:**

*Lactobacillales* including *L mesenteroides* have beneficial effects on human health, including improvement of psychological status and alleviation of allergic rhinitis. In mice, *L mesenteroides* subsp. strain NTM048 (NTM048) increased intestinal s-IgA. In humans, however, the effects of NTM048 on s-IgA secretion have been unclear.

**Study:**

This 16-week trial was performed using a double-blind, placebo-controlled, parallel group design. We aimed to establish whether *Leuconostoc mesenteroides* subsp. strain NTM048 increases the secretion of s-IgA in saliva. Forty healthy adults and forty patients with Japanese cedar pollinosis were recruited. Participants took either 2 test capsules including NTM048 (1010 CFU/day), or 2 placebo capsules per day, for 16 weeks. They were asked to collect their saliva and answered POMS2, a questionnaire about psychological status. The patients also answered questions about nasal symptoms. Blood samples were collected from the patients with Japanese Cedar pollinosis. Stool samples were collected at the start and on the last day of the trial.

**Results:**

All subjects completed the trial. It was conducted during the season when Japanese cedar pollen is most scattered. Serum concentration of Japanese cedar pollen-specific IgE was > 2.0 UA/mL in patients with Japanese cedar pollinosis. The amount of s-IgA in saliva was not increased by NTM048 in overall subjects, and Japanese cedar pollen-specific IgE was not changed by NTM048 in patients with Japanese cedar pollinosis. The symptom of nasal blockage was improved by NTM048 12 weeks after the start of trial. post hoc analysis indicated a positive correlation between improving psychological status and the increase in occupation ratio of lactobacillus including NTM048.

**Conclusion:**

The amount of s-IgA in saliva was not increased by NTM048, but nasal blockage was improved by it. Psychological status might be improved if dosage of NTM048 is raised to the degree that NTM048 might be increased in the intestinal tract.

## 1. Introduction

*Leuconostoc mesenteroides* is included within *Lactobacillales*, and several strains of *Lactobacillales* producing lactic acid have been shown to have beneficial effects on human health.^[1]^ *L mesenteroides* subsp. strain NTM048 (NTM048, Nitto Pharmaceutical Industries, Ltd., Kyoto, Japan) was isolated from green peas. In mice fed normal chow including NTM048 for up to 14 days, total secretory IgA (s-IgA) in stools was increased in a time-dependent manner.^[2]^ NTM048 produces exopolysaccharide (EPS), and the secretion of s-IgA in intestinal mucosa was increased in mice fed normal chow including EPS produced by NTM048, for 5 and 6 weeks.^[3]^ A part of the EPS produced by NTM048 promoted the production of IgA in mouse intestines.^[4]^ NTM048 may affect the secretion of s-IgA in human saliva, but this has not yet been demonstrated.

Japanese cedar pollen (JCP) is spread between February and April, with a peak in March.^[5]^ An epidemiological survey in 2001 indicated that the estimated prevalence of cedar pollen allergy was 13.1%.^[6]^ Cedar pollen allergy has been increasing in prevalence in Japan.^[5]^ Several probiotics are reportedly effective in the treatment of allergic inflammation,^[7]^ and previous clinical studies have indicated the effect of probiotics on Japanese cedar pollinosis. *Bifidobacterium longum* BB536 reportedly improved the symptoms of rhinorrhea and nasal blockage in patients with Japanese cedar pollinosis via the modulation of immune response, and it tended to suppress the elevation of JCP-specific IgE.^[8]^ In a clinical trial, *Lactobacillus* GG and *Lactobacillus gasseri* TMC0356 also improved the symptom of nasal blockage caused by Japanese cedar pollinosis via inhibition of IL4 and IL5 production.^[9]^ An element of the probiotics may therefore have the effect of improving the nasal symptoms of Japanese cedar pollinosis by change of immune response.

s-IgA prevents adherence and penetration of antigen including pollen through the mucosa.^[10]^ NTM048, which promotes s-IgA in mouse intestinal tracts, might improve the symptoms of JCP allergy by promoting the secretion of s-IgA. This study examines whether NTM048 promotes the secretion of s-IgA in saliva. To evaluate the effect of increasing s-IgA on penetration of JCP, we also examined whether NTM048 inhibits the elevating serum JCP-specific IgE level and improves the nasal symptoms of Japanese cedar pollinosis.

## 2. Materials and methods

### 2.1. Participants

We recruited Japanese adult men and women between 20 and 60 years old who could take capsules between November 20, 2017 and December 31, 2017 via Wakayama Medical University. Their eligibility was assessed according to the following exclusion criteria:

1) Symptoms of pollinosis other than cedar pollinosis at the time of screening (November 2017 to January 2018).
2) Subjects who regularly take probiotics (e.g., lactic acid bacteria beverages, food, and supplements, and/or *Bacillus subtilis* var. natto).
3) Smokers.
4) Pregnant women, lactating women, or women who want to become pregnant during the trial period.
5) Subjects who show hypersensitivity symptoms to lactic acid bacteria foods.
6) Subjects who doctors responsible for this study judge to be otherwise unsuitable for inclusion in the trial.

JCP-specific IgE levels were measured in all participants after informed consent to inclusion in this study was received. Participants whose JCP-specific serum IgE level was ≥ 0.7 U_A_/mL were considered to be patients with Japanese cedar pollinosis, while those < 0.7 U_A_/mL were considered to be healthy controls.

### 2.2. Study design

This sixteen-week trial was performed using a double-blind, placebo-controlled, parallel group design. Participants were asked to take 2 capsules per day as test foods for 16 weeks. The trial was started between January 25, 2018 and February 1, 2018. Participants visited Wakayama Medical University every 4 weeks (0, 4, 8, 12, and 16 weeks). The participants were asked to collect their saliva for 5 minutes with Salivette Cotton (Sarstedt, Nümbrecht, Germany) under an unstimulated condition. After collection of the saliva, all participants answered a questionnaire, *Profile of Mood States 2nd Edition* (POMS2; translated into Japanese), which was used in previous studies.^[11,12]^ The patients also answered a questionnaire on nasal symptoms (see Table S1, Supplemental Digital Content, http://links.lww.com/MD/K417), which is used in classification of the severity of symptoms of allergic rhinitis.^[13]^ Blood samples were collected only from the patients with Japanese Cedar pollinosis. Stool samples were collected at the start and on the last day of the trial. The study procedures were approved by the Wakayama Medical University Ethics Committee (approval number 2137) and registered as a University Hospital Medical Information Network Clinical Trials Registry (UMIN-CTR) clinical trial (unique trial number: UMIN000029968).

### 2.3. Sample size

Sample size was computed based on the primary end-point measurement of s-IgA level on the last day of trial. In a previous report, the difference of mean of s-IgA level between the start and end of trial was 52.2 in a *Lactobacillus plantarum* ONRICb0240 group and 21.8 in a placebo group, and standard deviation was 51.5.^[14]^ According to these results, 34 subjects per group were needed to achieve statistical power of 0.80, with a type I error of 0.05 for comparison of s-IgA level between the placebo and probiotics groups. Assuming that 20% of subjects would be excluded due to consent withdrawal or because of medication affecting bowel movement frequency, we planned enrollment of 40 subjects per group, making 80 subjects in total.

### 2.4. Measurement in blood and stool samples

Blood samples were centrifuged at 1500 g for 10 minutes, and concentrations of JCP-specific IgE in collected serum samples were measured by fluorescence enzyme immune assay (SRL, Tokyo, Japan). Stool samples were collected in spoon-type collection tubes and frozen at −80°C. Concentration of organic anions and short-chain fatty acids in stool samples were measured by liquid chromatography (Techno Suruga Co., Ltd., Shizuoka, Japan). Fecal bacteria were analyzed by Techno Suruga Co., Ltd. using the terminal restriction fragment length polymorphism-based method, according to methods used in a previous study.^[15]^ In brief, bacterial 16S DNA was amplified by PCR using fluorescently labeled primers. Amplified products were digested with restriction enzymes, and digested fragments were analyzed by electrophoresis. Table S2, http://links.lww.com/MD/K418 shows each length of fragment corresponding to bacterial species (see Table S2, Supplemental Digital Content, http://links.lww.com/MD/K418). The proportion of bacteria was calculated from the fluorescent intensity of each fragment.

#### 2.4.1. Test foods.

The test foods were capsules containing starch, calcium stearate and microcrystalline cellulose. Probiotic capsules also contained the viable cell count of *L mesenteroides* subsp. strain NTM048 (5 × 10^9^ colony-forming units (CFU)/capsule). All capsules were made from hydroxypropyl methylcellulose, and all test foods were produced by Genuine R&D (Fukuoka, Japan) according to good manufacturing practice. *L mesenteroides* subsp. strain NTM048 was supplied by Noster Inc. (Kyoto, Japan).

#### 2.4.2. Randomization and masking.

After obtaining written informed consent, we collected the age and sex of the participants. When the number of eligible participants reached 40 in both the healthy subjects and the patients with Japanese cedar pollinosis, these participants were enrolled.

In this study, a static allocation table adjusting sex and age (20–40/41–60) was made by the Clinical Study Support Center at Wakayama Medical University Hospital. Participant lists including test ID, subject status (healthy subjects/patients with Japanese cedar pollinosis), age and sex were sent to assignment staff in January 2018, who performed the allocation according to the table. The allocation results were then sent to a facilitator, who labeled the boxes containing test foods according to the results of allocation with ID for testing purposes. The individuals related to the allocation were not connected with the research staff, and all research staff and enrolled participants were unaware of the actual allocations. All data were fixed on October 9,2018, and the allocation key opening was performed. Data were analyzed by a statistician at the Clinical Study Support Center at Wakayama Medical University, according to the statistical analysis plan.

#### 2.4.3. Statistical analysis.

Primary outcome was the difference of s-IgA in saliva from start to end of the study (16 weeks after the start). Secondary outcomes were increase in s-IgA in saliva from the start to 4, 8, and 12 weeks after the start, increase in JCP-specific IgE in serum, each POMS2 score and the score of nasal symptoms 4, 8, 12, and 16 weeks after the start. Further secondary outcomes were increase in organic anions and fatty acid levels and the abundance ratio of each bacterium in fecal samples from the start to the end. The score of nasal symptoms and the abundance ratio of each bacterium in fecal samples were analyzed with Wilcoxon test, and other evaluated items were analyzed with Student *t* test. *P* < .05 was considered to be statistically significant.

To examine whether *L mesenteroides* subsp. strain NTM048 or other bacteria included in *Lactobacillales* were associated with the improvement of nasal blockage, post hoc tests were performed by regression analysis of the relation between increasing POMS2 score and increasing abundance ratio of *Lactobacillales* between the start and end of trial and logistic regression analysis on the relation between increasing nasal blockage score and increasing abundance ratio of *Lactobacillales* between the start and end of trial.

Data were analyzed using SAS 9.4 software (SAS Institute, Cary, NC) for primary and secondary outcomes and JMP Pro 14.1.0 software (SAS Institute) for post hoc analysis.

## 3. Results

### 3.1. Participants

**Figure 1.**
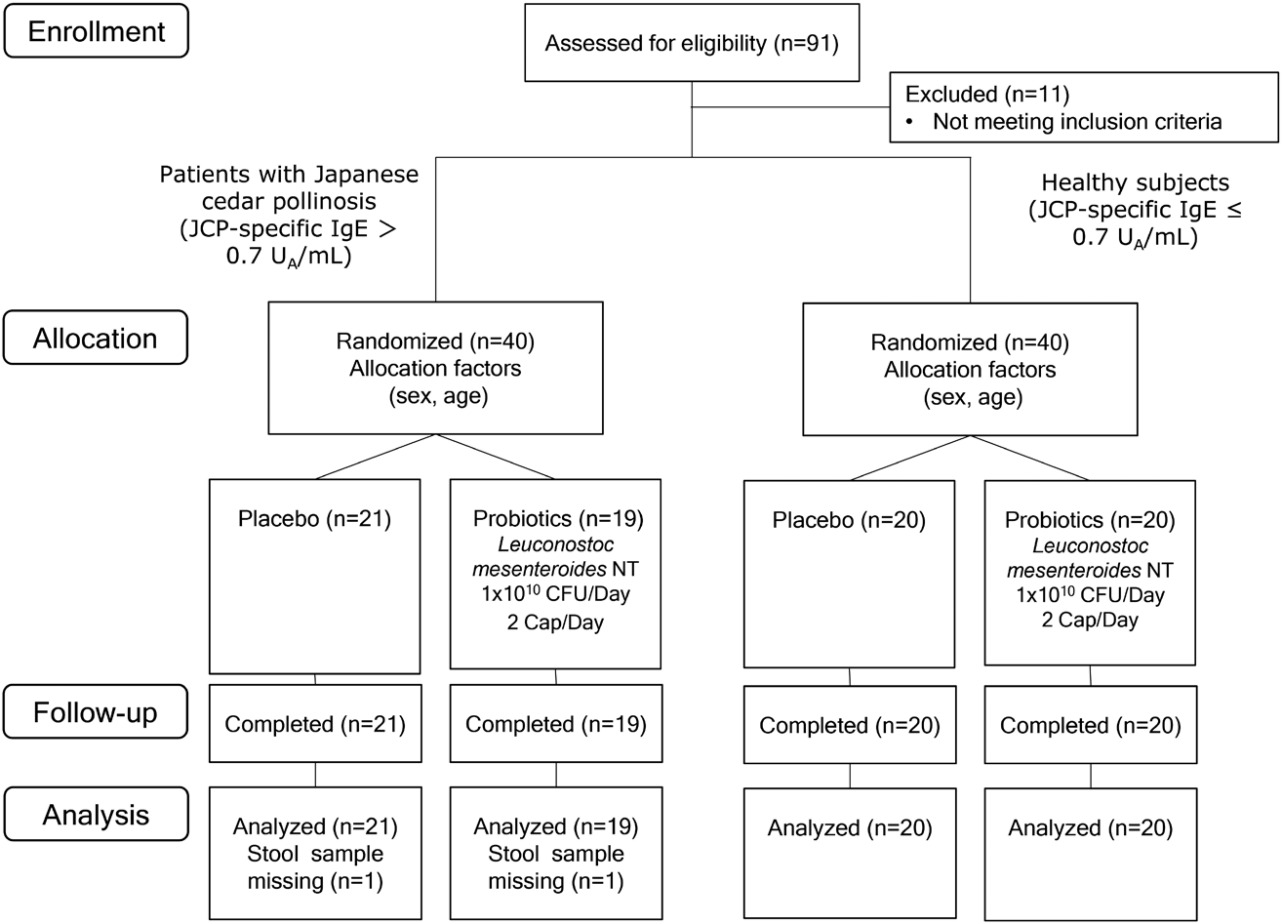
Flow chart of participant inclusion in this study.

Ninety-one people consented to inclusion after explanation of this study, all of whom lived nearby. Eleven people were excluded because they did not meet inclusion criteria. Forty healthy subjects were allocated to placebo (n = 21) and probiotics groups (n = 19), and forty patients with Japanese cedar pollinosis were allocated to placebo (n = 20) or probiotics groups (n = 20) (Fig. 1). Stool samples were not collected from all patients in the placebo and probiotics groups. Of the healthy subjects, more than 90% were female, and of the patients with Japanese cedar pollinosis, a little over 50% were female (Table 1). Serum concentration of JCP-specific IgE was > 2.0 UA/mL in patients with Japanese cedar pollinosis.

**Table 1:** Background of subjects.

	Control	Probiotics
All subjects		
n	41	39
Age (yr)	30.4 ± 10.0	30.1 ± 9.4
Female (%)	70.7	74.4
Healthy subjects		
n	21	19
Age (yr)	31.7 ± 10.5	33.2 ± 11.2
Female (%)	90.0	95.0
Patients with Japanese cedar pollinosis		
n	21	19
Age (yr)	29.2 ± 9.6	26.8 ± 5.8
Female (%)	52.4	52.6

### 3.2. Secretory IgA in saliva

We analyzed the primary outcome, difference of s-IgA in saliva at the end of the trial (16 w) from that at the start. s-IgA in saliva was not significantly increased in the probiotics group from the start to the end of the trial in comparison with the placebo group. As a secondary outcome, we also analyzed the difference of s-IgA in saliva from the start to 4, 8, and 12 weeks after the start of the trial. S-IgA secretion relative to baseline is shown in Table 2. There were no significant differences in s-IgA in saliva at any of the observation points, including comparison between that at the end of the trial (16 w) and that at the start. s-IgA in saliva at each observation point from the start were not changed by probiotics in either the healthy subjects or in patients with Japanese cedar pollinosis.

**Table 2:** s-IgA secretion (µg/5 min) (relative to baseline).

	Placebo		Probiotics		*P*
	Mean	95% CI	Mean	95% CI	
All subjects					
4 wk	−31.7	[−72.9, 9.4]	−21.5	[−67.6, 24.7]	.74
8 wk	−44.5	[−89.7, 0.7]	−51.5	[−95.2, −7.9]	.82
12 wk	−70.5	[−119.4, −21.6]	−81.7	[−128.5, −34.9]	.74
16 wk	14.8	[−44.1, 73.7]	34.9	[−22.7, 92.6]	.62
Healthy subjects					
4 wk	−50.1	[−118.0, 18.1]	14.9	[−66.5, 64.5]	.12
8 wk	−52.3	[−115.0, 9.9]	−13.1	[−95.2, 40.4]	.32
12 wk	−127.0	[−196.0, −57.9]	−79.1	[−126.0, −32.3]	.24
16 wk	19.3	[−90.9, 129.5]	81.7	[1.5, 161.9]	.34
Patients with Japanese cedar pollinosis					
4 wk	−14.2	[−66.5, 38.0]	−59.7	[−140.0, 20.8]	.32
8 wk	−37.0	[−108.0, 33.7]	−92.0	[−162.0, −22.0]	.26
12 wk	−16.8	[−82.9, 49.4]	−84.4	[−173.0, 3.7]	.20
16 wk	10.5	[−48.7, 69.8]	−14.3	[−98.8, 70.3]	.61

*P* values were calculated by Student *t* test.

### 3.3. Plasma JCP-specific IgE

In Japan, JCP are released into the air during the spring, and the peak of this release is in March and April. This trial was begun from late January to early February. The average JCP abundance in air was 4.1/m^3^/h in February, 13.2/m^3^/h in March, 11.4/m^3^/h in April and 6.6/m^3^/h in May (https://www.env.go.jp/page_00209.html).

Concentration of serum JCP-specific IgE relative to baseline are shown in Table 3. Plasma specific IgE against the JCP concentration had been increasing from the start to the end of the trial, and the increase of the plasma specific IgE against JCP was not lower in the probiotics group than in the placebo group at any of the observation points from the start.

**Table 3:** Concentration of serum JCP-specific IgE (relative to baseline).

	Placebo	Probiotics	95% CI	*P*
	Mean	Mean		
4 wk	−0.67	0.88	[−2.55, 5.66]	.45
8 wk	3.06	7.99	[−2.7, 12.56]	.20
12 wk	13.79	22.59	[−2.41, 20.02]	.12
16 wk	14.07	20.24	[−4.17, 16.51]	.23

95% CI indicated difference of IgE concentration between placebo and probiotics groups (Probiotics—Placebo). *P* values were calculated by Wilcoxon test.

### 3.4. Change of allergic rhinitis symptoms during JCP season

To evaluate improvement of allergic rhinitis symptoms in probiotics, we analyzed the change of scores in severity of allergic rhinitis symptoms from the start to all observation points. The severity of allergic rhinitis symptoms relative to baseline are shown in Table 4. Probiotics decreased the mean scores at 12 and 16 weeks in sneezing, rhinorrhea, nasal blockage and in troubles with daily life. However, only nasal blockage was significantly improved by probiotics at 12 weeks. There was no change to allergic rhinitis symptoms at 4 or 8 weeks.

**Table 4:** The severity of allergic rhinitis symptoms (relative to baseline).

	Placebo	Probiotics	95% CI	*P*
	Mean	Mean		
Paroxysmal sneezing (average number of episodes of paroxysmal sneezing in a day)				
4 wk	0.10	−0.26	[−0.17,0.88]	.30
8 wk	0.81	0.89	[−0.95,0.78]	.89
12 wk	0.19	−0.16	[−0.24,0.93]	.38
16 wk	−0.14	−0.53	[−0.07,0.84]	.11
Rhinorrhea (average number of episodes of nose blowing a day)				
4 wk	0.19	−0.05	[−0.25,0.73]	.48
8 wk	0.67	0.79	[−0.99,0.75]	.86
12 wk	0.29	−0.16	[−0.13,1.02]	.24
16 wk	0.05	−0.32	[−0.16,0.88]	.21
Nasal blockage				
4 wk	0.05	0.05	[−0.49,0.48]	.95
8 wk	0.57	0.47	[−0.41,0.61]	.66
12 wk	0.48	−0.11	[0.06,1.10]	.05*
16 wk	0.00	−0.26	[−0.26,0.79]	.22
Troubles with daily life				
4 wk	0.05	−0.26	[−0.21,0.83]	.23
8 wk	0.38	0.37	[−0.61,0.63]	.98
12 wk	0.19	−0.05	[−0.22,0.71]	.39
16 wk	−0.10	−0.37	[−0.17,0.72]	.17

95% CI indicated difference of each nasal symptoms score between placebo and probiotics groups (Probiotics—Placebo). *P* values were calculated by exactly Wilcoxon test. An asterisk indicates that *P* < .05.

### 3.5. Effect of probiotics on intestinal flora

To evaluate the effect of probiotics in intestinal flora, we analyzed the fatty acids and organic anions, which were metabolites of intestinal bacteria. Short-chain fatty acids and organic anions in stools relative to baseline are shown in Table 5. No fatty acids or lactic acid were changed by probiotics in stools of any subjects, but succinic acid was significantly increased in the probiotics group. The profile of fatty acids and organic anions in stools of patients with Japanese cedar pollinosis were similar in stools of all subjects. Change of succinic acids in stools was not observed in healthy subjects.

**Table 5:** Short-chain fatty acid and organic anions in stool (relative to baseline).

	Placebo	Probiotics	95% CI	*P*
	Mean	Mean		
All subjects				
Succinic acid	0.09	0.22	[−0.12, 0.39]	.03*
Lactic acid	−0.25	0.01	[−0.05, 0.58]	.62
Formic acid	−0.01	0.00	[−0.01, 0.03]	.65
Acetic acid	0.03	0.21	[−0.40, 0.77]	.90
Propionic acid	0.03	0.05	[−0.25, 0.29]	.94
Iso-butyric acid	0.00	0.01	[−0.04, 0.06]	.92
n-butyric acid	−0.02	0.09	[−0.13, 0.34]	.61
Iso-pentanoic acid	−0.02	0.02	[−0.05, 0.12]	.29
n-pentanoic acid	−0.01	−0.02	[−0.06, 0.05]	.88
Healthy subjects				
Succinic acid	0.06	0.08	[−0.17, 0.21]	.31
Lactic acid	−0.03	−0.02	[−0.10, 0.12]	.43
Formic acid	−0.01	−0.01	[−0.02, 0.03]	.97
Acetic acid	−0.05	0.27	[−0.54, 1.17]	.77
Propionic acid	−0.07	0.02	[−0.25, 0.42]	.48
Iso-butyric acid	0.00	0.00	[−0.08, 0.08]	.79
n-butyric acid	−0.01	0.07	[−0.26, 0.43]	.87
Iso-pentanoic acid	−0.03	0.03	[−0.06, 0.18]	.22
n-pentanoic acid	0.00	−0.04	[−0.12, 0.05]	.54
Patients with Japanese cedar pollinosis				
Succinic acid	0.12	0.39	[−0.23, 0.77]	.05*
Lactic acid	−0.48	0.05	[−0.11, 1.17]	.21
Formic acid	0.00	0.01	[−0.02, 0.04]	.53
Acetic acid	0.11	0.16	[−0.81, 0.91]	.95
Propionic acid	0.13	0.08	[−0.49, 0.40]	.64
Iso-butyric acid	−0.01	0.01	[−0.05, 0.09]	.67
n-butyric acid	−0.02	0.10	[−0.21, 0.45]	.43
Iso-pentanoic acid	−0.01	0.00	[−0.12, 0.14]	.86
n-pentanoic acid	−0.02	0.01	[−0.06, 0.11]	.59

95% CI indicated difference of fatty acids and organic anions concentration between placebo and probiotics groups (Probiotics—Placebo). *P* values were calculated by Wilcoxon test. Asterisks indicate that *P* < .05.

To confirm the increase of *Lactobacillales* including NTM048 in the stools of the probiotics group, we analyzed the profile of intestinal flora in stool samples. The occupation ratios of microbacteria in stools are shown in Table 6. The occupation ratio of *Lactobacillales* was significantly increased in the probiotics group, and it tended to be increased in the probiotics group in both healthy subjects and in patients with Japanese cedar pollinosis.

**Table 6:** Occupation ratio (relative to baseline) of microbacteria in stool.

	Placebo	Probiotics	95% CI	*P*
	Mean	Mean		
All subjects				
*Bifidobacterium*	0.91	0.59	[−5.24, 4.59]	.89
*Lactobacillales*	−0.43	1.47	[−1.14, 4.94]	.05*
*Bacteroides*	0.74	−1.82	[−9.42, 4.29]	.55
*Prevotella*	0.36	−0.01	[−1.01, 0.27]	.52
*Clostridium*				
*Clostridium cluster IV*	0.06	1.50	[−0.58, 3.46]	.20
*Clostridium cluster IX*	−0.18	−1.14	[−4.43, 2.52]	.29
*Clostridium cluster XI*	−0.36	−0.20	[−0.82, 1.13]	.31
*Clostridium subcluster XIVa*	−1.29	−0.10	[−2.48, 4.86]	.25
*Clostridium cluster XVIII*	0.16	−0.17	[−1.02, 0.35]	.50
*Other bacteria*	0.03	−0.10	[−1.02, 0.35]	.46
Healthy subjects				
*Bifidobacterium*	0.78	3.81	[−0.30, 4.24]	.50
*Lactobacillales*	−0.13	1.42	[−6.35, 3.24]	.15
*Bacteroides*	2.49	−7.27	[−0.49, 20.02]	.11
*Prevotella*	0.18	−0.19	[−0.09, 0.71]	.16
*Clostridium*				
*Clostridium cluster IV*	0.74	2.25	[−4.61, 1.59]	.45
*Clostridium cluster IX*	0.48	0.25	[−3.66, 5.11]	.89
*Clostridium cluster XI*	0.05	−0.17	[−0.55, 0.98]	.35
*Clostridium subcluster XIVa*	−2.91	1.02	[−8.76, 0.90]	.13
*Clostridium cluster XVIII*	0.15	−0.36	[−0.61, 1.64]	.26
*Other bacteria*	−1.83	−0.32	[−5.03, 2.01]	.68
Patients with Japan cedar pollinosis				
*Bifidobacterium*	1.04	−3.00	[−2.62, 10.72]	.27
*Lactobacillales*	−0.73	1.52	[−6.22, 1.72]	.27
*Bacteroides*	−1.01	4.24	[−4.05, 3.56]	.35
*Prevotella*	0.53	0.12	[−0.88, 1.70]	.74
*Clostridium*				
*Clostridium cluster IV*	−0.62	0.66	[−3.94, 1.38]	.37
*Clostridium cluster IX*	−0.84	−2.11	[−4.40, 6.95]	.19
*Clostridium cluster XI*	−0.76	−0.24	[−2.40, 1.36]	.66
*Clostridium subcluster XIVa*	0.32	−1.35	[−4.04, 7.39]	.98
*Clostridium cluster XVIII*	0.17	0.03	[−0.67, 0.95]	.70
*Other bacteria*	1.89	0.14	[−1.34, 4.85]	.11

95% CI indicated difference of occupation ratio of microbacteria between placebo and probiotics groups (Probiotics—Placebo). *P* values were calculated by Wilcoxon test. Asterisks indicate that *P* < .05.

### 3.6. Effect of probiotics on psychological conditions

We monitored the psychological conditions of all subjects in the trial using the POMS2 questionnaire to evaluate the condition of anger-hostility (AH), confusion-bewilderment (CB), depression-dejection (DD), fatigue-inertia (FI), tension-anxiety (TA), vigor-activity (VA), friendliness (F), and total mood disturbances (TMD). POMS2 t-scores relative to baseline are shown in Table 7. VA was significantly worsened in the probiotics group 8 weeks after the start, and the tendency was observed 12 and 16 weeks after the start. FI tended to ameliorate in the probiotics group 8 and 12 weeks from the start, but the other scores were not changed by NTM048.

**Table 7:** POMS2 t-scores (relative to baseline).

	Placebo	Probiotics	95% CI	*P*
	Mean	Mean		
TMD				
4 wk	−0.41	−0.21	[−2.26, 2.68]	.87
8 wk	0.05	0.56	[−1.49, 2.52]	.61
12 wk	0.56	−0.64	[−3.96, 1.56]	.39
16 wk	−1.07	0.21	[−2.01, 4.56]	.44
AH				
4 wk	1.54	0.10	[−4.83, 1.96]	.40
8 wk	1.29	0.36	[−3.65, 1.78]	.50
12 wk	1.66	0.67	[−4.24, 2.26]	.55
16 wk	−0.27	0.49	[−2.45, 3.96]	.64
CB				
4 wk	−0.44	−0.82	[−3.00, 2.24]	.77
8 wk	−1.05	−0.23	[−1.38, 3.01]	.46
12 wk	−0.15	−1.90	[−4.40, 0.89]	.19
16 wk	−1.17	−0.67	[−2.27, 3.28]	.72
DD				
4 wk	0.32	0.18	[−2.42, 2.15]	.90
8 wk	0.54	1.54	[−1.28, 3.28]	.38
12 wk	1.05	−0.21	[−0.81, 1.30]	.33
16 wk	0.41	1.08	[−2.74, 4.07]	.70
FI				
4 wk	0.05	−0.62	[−3.80, 2.48]	.67
8 wk	0.85	−1.62	[−5.09, 0.15]	.06
12 wk	1.17	−1.79	[−6.27, 0.34]	.08
16 wk	−0.68	−1.90	[−4.71, 2.28]	.49
TA				
4 wk	−2.56	−0.67	[−1.25, 5.04]	.23
8 wk	−1.17	−0.69	[−2.85, 3.80]	.78
12 wk	0.29	−1.90	[−5.76, 1.38]	.23
16 wk	−2.54	−1.03	[−2.47, 5.49]	.45
VA				
4 wk	1.41	−0.10	[−4.25, 1.21]	.27
8 wk	0.49	−2.33	[−0.55, −0.09]	.04*
12 wk	2.00	−1.10	[−6.22, 0.02]	.05
16 wk	1.54	−1.51	[−6.37, 0.27]	.07
F				
4 wk	0.34	−0.79	[−4.25, 1.97]	.47
8 wk	−1.83	−2.74	[−4.09, 2.27]	.57
12 wk	−0.59	−2.69	[−5.65, 1.44]	.24
16 wk	−1.71	−2.79	[−4.30, 2.13]	.50

95% CI indicated difference of t-scores (relative to baseline) in each variable between placebo and probiotics groups (Probiotics—Placebo). *P* values were calculated by Student test. Asterisks indicate that *P* < .05.

AH = anger- hostility, CB = confusion-bewilderment, DD = depression- dejection, F = Friendliness, FI = fatigue-inertia, TA = tension-anxiety, TMD = total mood disturbance, VA = vigor-activity.

### 3.7. Post hoc analysis in relation to occupation ratio to *Lactobacillales*

**Figure 2.**
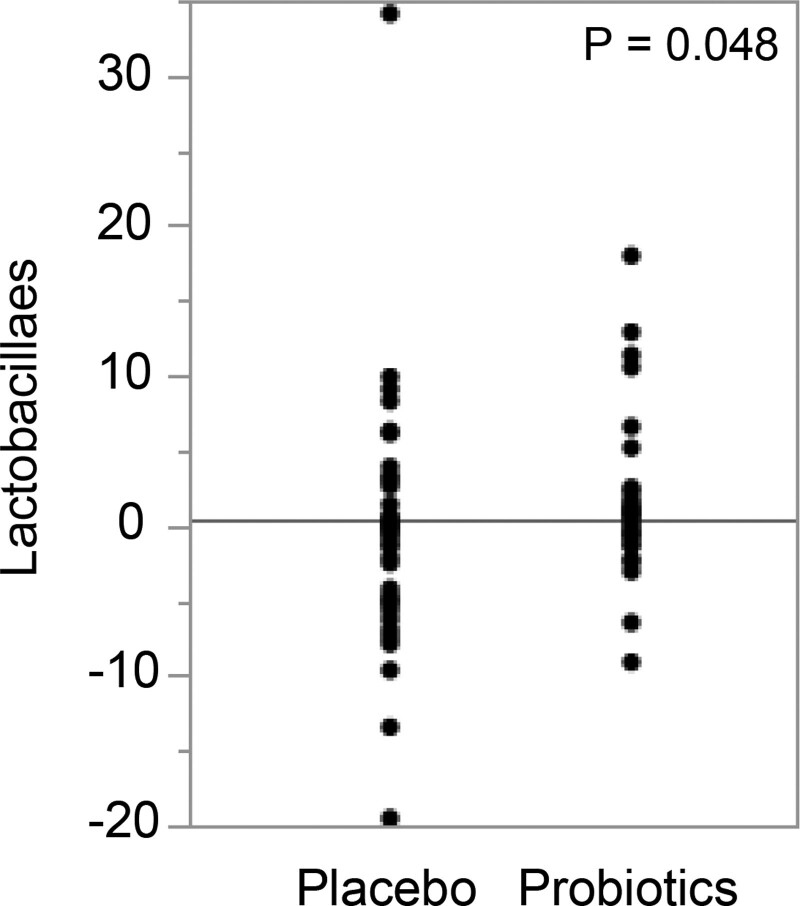
Changing occupation ratio of *Lactobacillales* in this study. The occupation ratio (relative to baseline) of *Lactobacillales* in each subject is plotted with black dots.

Analysis of intestinal flora indicated that the occupation ratio of *Lactobacillales* including NTM048 was significantly increased in the probiotics group, but the variance of the occupation ratio was wide in both of the groups, especially in the placebo group (Fig. 2). In probiotics group, we examined the significance of the correlation between the changes of s-IgA, POMS2 score and nasal symptom score with increase in the occupation ratio of *Lactobacillales* in intestinal flora. We hypothesized that the changes of s-IgA, POMS2 score and nasal symptom score would be improved by increase in the occupation ratio of *Lactobacillales* resulting from administration of NTM048. Regarding the primary outcome of this study, s-IgA was not increased by NTM048. We further analyzed the relation between increase in occupation ratio of *Lactobacillales* by NTM048 and increase in secretion of s-IgA in saliva. However, the correlation between occupation ratio of *Lactobacillales* and the amount of s-IgA in saliva in the probiotics group was also not significant (see Fig. S1, Supplemental Digital Content, http://links.lww.com/MD/K419). Meanwhile, aspects of psychological status were significantly changed or tended to be changed by NTM048 (Table 7), but the changes of mental status were not consistent in the probiotics group. For clarification, we analyzed the correlations between the changes of POMS2 scores and increase in the occupation ratio of *Lactobacillales* in all subjects and in the probiotics group alone. TMD and each status was not significantly correlated with the occupation ratio of *Lactobacillales* in any of the subjects (see Fig. S2, Supplemental Digital Content, http://links.lww.com/MD/K420). The scores of TMD, DD, and FI, which had negative correlation with positive psychological status, had negative correlation to increase in the occupation ratio of *Lactobacillales* in the probiotics group only (see Fig. S2, Supplemental Digital Content, http://links.lww.com/MD/K420). The scores of VA and F, which had positive correlation with positive psychological status, had positive correlation with increase in the occupation ratio of *Lactobacillales* in the probiotics group only (see Fig. S2, Supplemental Digital Content, http://links.lww.com/MD/K420).

In this study, the symptom of nasal blockage was improved by NTM048 12 weeks after the start of trial. The changes of occupation ratio were affected by NTM048 for 16 weeks. The changes of occupation ratio could therefore be directly reflected in improvement of nasal blockage 12 weeks after the start of the trial. Hypothesizing that the occupation ratio was linearly increased in a time-dependent manner, we evaluated the association between improvement of nasal blockage and increasing the occupation ratio of *Lactobacillales*. The occupation ratio of subjects whose nasal blockage was improved was plotted in green dots and the occupation ratio of subjects whose nasal blockage was aggravated was plotted in red dots (see Fig. S3, Supplemental Digital Content, http://links.lww.com/MD/K421). The occupation ratio of *Lactobacillales* was increased in the flora of patients whose nasal blockage was improved in the probiotics group, but not in the placebo group. Logistic regression analysis was performed because the increase in occupation ratio of *Lactobacillales* by the oral administration of NTM048 might be associated with improvement of the symptoms of nasal blockage. Logistic regression analysis showed that the increase in occupation ratio of *Lactobacillales* was significantly associated with improvement of the symptoms of nasal blockage in all patients, not only those in the probiotics group.

## 4. Discussion

This randomized, double-blinded, placebo-controlled trial examined whether a probiotic, NTM048, increased the amount of s-IgA in saliva of adults who took an oral dose for 16 weeks during the period when JCP are most scattered in the air in Japan. In the current study, there was no promotion of secretion of s-IgA in saliva in the probiotics group at 4, 8, 12 and 16 weeks after the start of the trial (Table 2). NTM048 did not inhibit the increase of JCP-specific IgE in serum of Japan cedar pollinosis (Table 3). Nasal blockage was, however, improved by NTM048 12 weeks after the start of trial, when the amount of JCP in the air was nearly at its peak (Table 4). NTM048 also increased the concentration of succinic acid and the abundance ratio of *Lactobacillales* in fecal samples (Table 5). Mental status was not consistently changed according to POMS score (Table 7).

s-IgA is the most abundant immunoglobulin isotype in saliva, and s-IgA protects invasion of microorganisms in oral mucosa.^[16]^ In the current study, subjects were allocated with adjustment for sex and age because the amount of s-IgA in saliva is higher in women than in men,^[17,18]^ and it is higher in younger than in older individuals.^[19]^ The bias associated with secretion of s-IgA in saliva was thought to be minimized in this study. s-IgA in saliva was not promoted by NTM048 in overall subjects, healthy subjects, or in patients with Japanese cedar pollinosis (Table 2). In previous studies, the amount of s-IgA in saliva was increased by *Lactobacillus paracasei* SD1 for 12 weeks in children,^[20]^ but it was not changed by *Lactobacillus reuteri* for 3 weeks in healthy adults.^[21]^ The promotion of the secretion of s-IgA in saliva may be dependent on the strains of probiotics used. Non-clinical tests indicated that intestinal s-IgA was increased in mice fed normal chow including NTM048 for 14 days,^[2]^ but there was no increase in s-IgA in saliva. NTM048 did not promote the secretion of s-IgA in saliva in this study, but it remains to be seen whether NTM048 promotes s-IgA in the intestine.

Probiotics reportedly prevented the IgE-associated allergy including eczema.^[22,23]^ *B longum* BB536 improved nasal blockage and rhinorrhea scores without inhibiting the elevation of JCP-specific IgE.^[8]^ In the current study, NTM048 also improved nasal blockage in Japanese cedar pollinosis without inhibition of elevation of JCP-specific IgE (Table 3). *B longum* BB536 decreased the level of IL-10 and the eosinophil rate in the blood, as well as improving nasal blockage and rhinorrhea scores.^[8]^ NTM048 may also improve the symptom of nasal blockage by modulation of the function of cytokine secretions via TH2 and Treg cells, but we did not measure the profile of cytokines in the blood this time. To better understand the mechanisms of the improving nasal symptoms, future probiotics studies must measure the profile of cytokines and the eosinophil rate in peripheral blood.

Psychological status was measured by POMS2 following collection of saliva samples, because salivary s-IgA was reported to be affected by acute psychological stress.^[24]^ The current study indicated that neither secretion of s-IgA nor score of psychological status were changed without VA (Table 2 and Table 7). High dose of *Lactiplantibacillus plantarum* SNK12 (3 × 10^10^ CFU/day) was reported to have led to greater improvement of psychological status than low dose (1 × 10^10^ CFU/day).^[25]^ The dose of this study was only 1 × 10^10^ CFU/day. This could be problematic because a part of the probiotics taken orally is killed in gastric juice and the amount of surviving probiotics would not reveal the sufficient effects.^[26]^ post hoc correlation analysis indicated that the change in all scores of POMS2 was not due to change in occupation ratio of *Lactobacillales* in overall subjects. TMD, DD and FI, which reflect upon negative psychological status^[27]^ were, however, negatively correlated with the change in occupation ratio of *Lactobacillales* in the probiotics group (see Fig. S2, Supplemental Digital Content Furthermore, http://links.lww.com/MD/K420, VA and F, which reflect positive psychological status,^[27]^ were positively correlated with the change in occupation ratio of *Lactobacillales* in the probiotics group (see Fig. S2, Supplemental Digital Content, http://links.lww.com/MD/K420). Psychological status might therefore be improved if NTM048 could reach the intestinal tract to a sufficient level.

NTM048 increased the level of succinic acid in fecal samples of all subjects or in samples of patients with Japanese cedar pollinosis (Table 5). To detect the microorganisms associated with elevating succinic acid in fecal samples, we searched the microorganisms that had an occupation ratio increased by NTM048 in all subjects and in patients with Japanese cedar pollinosis. There were, however, no organisms matched for the condition. Succinic acid is produced and consumed by several bacterial species, but there is no standard classification for succinic acid producers or consumers.^[28]^ The mechanism behind elevation of succinic acid in the stool was therefore unclear. Elevation of the level of succinic acid but not lactic acid may indicate the possibility of an insufficient amount of live NTM048 in the small intestine.

This study demonstrated that NTM048 strain ameliorated the nasal blockage in the 12 weeks when JCP was most scattered in the air. Focusing on the relationship between the improvement of nasal blockage and occupation ratio of *Lactobacillales* in stools, dot plots show aggravated patients in red and improved patients in green (Fig. S3A, Supplemental Digital Content, http://links.lww.com/MD/K421). The occupation ratio of *Lactobacillales* was increased by NTM048 but not by placebo in the stools of patients with improvement of nasal blockage (see Fig. S3A, Supplemental Digital Content, http://links.lww.com/MD/K421). Logistic analysis was performed to analyze the relationship between increase in the occupation ratio of *Lactobacillales* and improvement of nasal blockage in overall patients and in the probiotics group (see Fig. S3B and C, Supplemental Digital Content, http://links.lww.com/MD/K421). The relationship was significant in overall patients, but not in the probiotics group. The disappearance of significant correlation was thought to be caused by there being too few patients for performance of logistic analysis. Decrease in the occupation ratio of *Lactobacillales* might therefore be associated with worsening of the symptom of nasal blockage, and the occupation ratio of *Lactobacillales* increased by oral administration of NTM048 might improve the symptom of nasal blockage.

This study was performed to examine whether NTM048 promoted the secretion of total s-IgA in saliva, and whether any promotion improved Japanese cedar pollinosis. Due to this goal, healthy subjects and patients with Japanese cedar pollinosis were recruited. NTM048 could not promote secretion of total s-IgA in the saliva of either group of adult subjects. The mechanisms of improving nasal blockage in NTM048 administration remain unclear in the current study because blood cytokine levels and cytometric profiling of whole blood were not measured. Adequate dose of NTM048 to improve the psychological status was unclear because this dose (1 × 10^10^ CFU/day) may not be able to increase the number of NTM048 in the intestine of all subjects taking probiotics and thus not improve the psychological status in the probiotics group. Further studies must examine whether nasal blockage and psychological status is improved by NTM048 in a dose-dependent manner, and quantitative real-time PCR analysis is also needed to measure the amount of NTM048 in the stool.

In conclusion, NTM048 did not promote secretion of s-IgA in saliva. However, it did improve the symptom of nasal blockage associated with JCP. post hoc study indicated that the psychological status was improved in the subjects with possible increase in occupation NTM048 ratio in the stool. Future study should analyze the improvement of specific psychological status and the symptom of nasal blockage in a dose-dependent manner.

## Acknowledgments

We acknowledge editing and proofreading by Benjamin Phillis from the Clinical Study Support Center at Wakayama Medical University.

## Author contributions

**Conceptualization:** Yuta Yamamoto.

**Data curation:** Yuta Yamamoto, Naoko Yamagishi, Shuji Kawashima, Toshio Nishi, Sachi Yamamoto.

**Formal analysis:** Kensuke Tanioka.

**Investigation:** Yuta Yamamoto, Gen Sugita, Masanobu Hiraoka, Saori Takagi.

**Project administration:** Yuta Yamamoto.

**Supervision:** Chiemi Kakutani, Agi Yanase, Yoshimitsu Kanai, Seiya Kato, Muneki Hotomi.

**Writing – original draft:** Yuta Yamamoto.

## Supplementary Material











## References

[1] De Filippis F Pasolli E Ercolini D. The food-gut axis: lactic acid bacteria and their link to food, the gut microbiome and human health. FEMS Microbiol Rev. 2020;44:454–89. 32556166 10.1093/femsre/fuaa015 PMC7391071

[2] Matsuzaki C Kamishima K Matsumoto K. Immunomodulating activity of exopolysaccharide-producing *Leuconostoc mesenteroides* strain NTM048 from green peas. J Appl Microbiol. 2014;116:980–9. 24314091 10.1111/jam.12411

[3] Matsuzaki C Hayakawa A Matsumoto K. Exopolysaccharides produced by *Leuconostoc mesenteroides* strain NTM048 as an immunostimulant to enhance the mucosal barrier and influence the systemic immune response. J Agric Food Chem. 2015;63:7009–15. 26207929 10.1021/acs.jafc.5b01960

[4] Matsuzaki C Nakashima Y Endo I. Enzymatically synthesized exopolysaccharide of a probiotic strain *Leuconostoc mesenteroides* NTM048 shows adjuvant activity to promote IgA antibody responses. Gut Microbes. 2021;13:1949097. 34288820 10.1080/19490976.2021.1949097 PMC8550178

[5] Osada T Okano M. Japanese cedar and cypress pollinosis updated: new allergens, cross-reactivity, and treatment. Allergol Int. 2021;70:281–90. 33962864 10.1016/j.alit.2021.04.002

[6] Okuda M. Epidemiology of Japanese cedar pollinosis throughout Japan. Ann Allergy Asthma Immunol. 2003;91:288–96. 14533662 10.1016/S1081-1206(10)63532-6

[7] Chang CJ Lin TL Tsai YL. Next generation probiotics in disease amelioration. J Food Drug Anal. 2019;27:615–22. 31324278 10.1016/j.jfda.2018.12.011 PMC9307044

[8] Xiao JZ Kondo S Yanagisawa N. Probiotics in the treatment of Japanese cedar pollinosis: a double-blind placebo-controlled trial. Clin Exp Allergy. 2006;36:1425–35. 17083353 10.1111/j.1365-2222.2006.02575.x

[9] Kawase M He F Kubota A. Effect of fermented milk prepared with two probiotic strains on Japanese cedar pollinosis in a double-blind placebo-controlled clinical study. Int J Food Microbiol. 2009;128:429–34. 18977549 10.1016/j.ijfoodmicro.2008.09.017

[10] Cerutti A Chen K Chorny A. Immunoglobulin responses at the mucosal interface. Annu Rev Immunol. 2011;29:273–93. 21219173 10.1146/annurev-immunol-031210-101317 PMC3064559

[11] KobayashiYKinoshitaTMatsumotoA. Bifidobacterium breve a1 supplementation improved cognitive decline in older adults with mild cognitive impairment: an open-label, single-arm study. J Prev Alzheimers Dis. 2019;6:70–5.3056908910.14283/jpad.2018.32

[12] Lee S Yamamoto S Kumagai-Takei N. Didgeridoo health promotion method improves mood, mental stress, and stability of autonomic nervous system. Int J Environ Res Public Health. 2019;16:3443. 31533214 10.3390/ijerph16183443 PMC6765776

[13] Okubo K Kurono Y Ichimura K. Japanese guidelines for allergic rhinitis 2020. Allergol Int. 2020;69:331–45. 32473790 10.1016/j.alit.2020.04.001

[14] Kishi K Kotani Y Yamahira S. *Lactobacillus plantarum* ONRICb0240 enhanced salivary IgA in healthy adult volunteers. Jpn J Lactic Acid Bact. 2006;17:132–7.

[15] Mizuno H Bamba S Abe N. Effects of an alginate-containing variable-viscosity enteral nutrition formula on defecation, intestinal microbiota, and short-chain fatty acid production. J Funct Foods. 2020;67:103852.

[16] Gong W Qiao Y Li B. The alteration of salivary immunoglobulin A in autism spectrum disorders. Front Psychiatry. 2021;12:669193. 34093280 10.3389/fpsyt.2021.669193 PMC8175640

[17] Jafarzadeh A Sadeghi M Karam GA. Salivary IgA and IgE levels in healthy subjects: relation to age and gender. Braz Oral Res. 2010;24:21–7. 20339709 10.1590/s1806-83242010000100004

[18] Birkett M Johnson L Gelety C. Investigation of sex differences in sIgA response to the trier social stress test. Stress Health. 2017;33:158–63. 27075235 10.1002/smi.2680

[19] Miletic ID Schiffman SS Miletic VD. Salivary IgA secretion rate in young and elderly persons. Physiol Behav. 1996;60:243–8. 8804670 10.1016/0031-9384(95)02161-2

[20] Pahumunto N Sophatha B Piwat S. Increasing salivary IgA and reducing Streptococcus mutans by probiotic *Lactobacillus paracasei* SD1: a double-blind, randomized, controlled study. J Dent Sci. 2019;14:178–84. 31210892 10.1016/j.jds.2019.01.008 PMC6562187

[21] Jorgensen MR Keller MK Kragelund C. *Lactobacillus reuteri* supplements do not affect salivary IgA or cytokine levels in healthy subjects: a randomized, double-blind, placebo-controlled, cross-over trial. Acta Odontol Scand. 2016;74:399–404. 27104984 10.3109/00016357.2016.1169439

[22] Abrahamsson TR Jakobsson T Bottcher MF. Probiotics in prevention of IgE-associated eczema: a double-blind, randomized, placebo-controlled trial. J Allergy Clin Immunol. 2007;119:1174–80. 17349686 10.1016/j.jaci.2007.01.007

[23] Kuitunen M Kukkonen K Juntunen-Backman K. Probiotics prevent IgE-associated allergy until age 5 years in cesarean-delivered children but not in the total cohort. J Allergy Clin Immunol. 2009;123:335–41. 19135235 10.1016/j.jaci.2008.11.019

[24] Takanari J Nakahigashi J Sato A. Effect of Enzyme-Treated Asparagus Extract (ETAS) on psychological stress in healthy individuals. J Nutr Sci Vitaminol (Tokyo). 2016;62:198–205. 27465727 10.3177/jnsv.62.198

[25] Watanabe T Hayashi K Takara T. Effect of oral administration of Lactiplantibacillus plantarum SNK12 on temporary stress in adults: a randomized, placebo-controlled, double-blind, parallel-group study. Int J Environ Res Public Health. 2022;19:8936. 35897310 10.3390/ijerph19158936 PMC9332698

[26] Cook MT Tzortzis G Charalampopoulos D. Microencapsulation of probiotics for gastrointestinal delivery. J Control Release. 2012;162:56–67. 22698940 10.1016/j.jconrel.2012.06.003

[27] Higashikawa F Kanno K Ogata A. Reduction of fatigue and anger-hostility by the oral administration of 5-aminolevulinic acid phosphate: a randomized, double-blind, placebo-controlled, parallel study. Sci Rep. 2020;10:16004. 32994490 10.1038/s41598-020-72763-4 PMC7525460

[28] Fernandez-Veledo S Vendrell J. Gut microbiota-derived succinate: friend or foe in human metabolic diseases? Rev Endocr Metab Disord. 2019;20:439–47. 31654259 10.1007/s11154-019-09513-z PMC6938788

