# Supplementary material for: Leuconostoc mesenteroides subsp. strain NTM048 ameliorated nasal symptoms in patients with Japan cedar pollinosis: Randomized, double-blind, and placebo-controlled trial
Source: Medicine (Baltimore). 2023 Nov 10;102(45):e35343. doi: 10.1097/MD.0000000000035343 (PMC10637569; doi:10.1097/MD.0000000000035343)
Supplement: Supplementary file 1 [file medi-102-e35343-s001.docx]

Table S1. Questionnaire about severity of allergic rhinitis symptoms

|  | Severity | | | | |
| --- | --- | --- | --- | --- | --- |
|  | 4 | 3 | 2 | 1 | 0 |
| Paroxysmal sneezing (average number of episodes of paroxysmal sneezing/day) | ≥ 21 times | 20-11 times | 10-6 times | 5-1 times | None |
| Rhinorrhea (average number of episodes of nose blowing/day | ≥ 21 times | 20-11 times | 10-6 times | 5-1 times | None |
| Nasal blockage | Completely obstructed all day | Severe nasal blockage causing prolonged oral breathing in a day | Severe nasal blockage causing occasional oral breathing in a day | Nasal blockage without oral breathing | None |
| Troubles with daily life | Impossible | Painful and complicating daily life | Intermediate between (3) and (1) | Few troubles | None |
