# Supplementary material for: Leuconostoc mesenteroides subsp. strain NTM048 ameliorated nasal symptoms in patients with Japan cedar pollinosis: Randomized, double-blind, and placebo-controlled trial
Source: Medicine (Baltimore). 2023 Nov 10;102(45):e35343. doi: 10.1097/MD.0000000000035343 (PMC10637569; doi:10.1097/MD.0000000000035343)
Supplement: Supplementary file 2 [file medi-102-e35343-s002.docx]

Table S2. Correspondence between fragments and phylogenetic bacterial groups

| Phylogenetic bacterial groups | Length of fragments (bp) |
| --- | --- |
| *Bifidobacterium* | 124 |
| *Lactobacillales* | 332, 517, 657 |
| *Prevotella* | 144 |
| *Bacteroides* | 366, 469, 853 |
| *Clostridium subcluster XIVa* | 106, 494, 505, 517, 754 919, 940, 955, 990 |
| *Clostridium cluster IX* | 110 |
| *Clostridium cluster IV* | 168, 369, 749 |
| *Clostridium cluster XI* | 919 |
| *Clostridium cluster XVIII* | 423, 650 |
| other bacteria | 443, 550, 770, 968 |
