# Supplementary material for: Leuconostoc mesenteroides subsp. strain NTM048 ameliorated nasal symptoms in patients with Japan cedar pollinosis: Randomized, double-blind, and placebo-controlled trial
Source: Medicine (Baltimore). 2023 Nov 10;102(45):e35343. doi: 10.1097/MD.0000000000035343 (PMC10637569; doi:10.1097/MD.0000000000035343)
Supplement: Supplementary file 3 [file medi-102-e35343-s003.docx]

Figure S1.


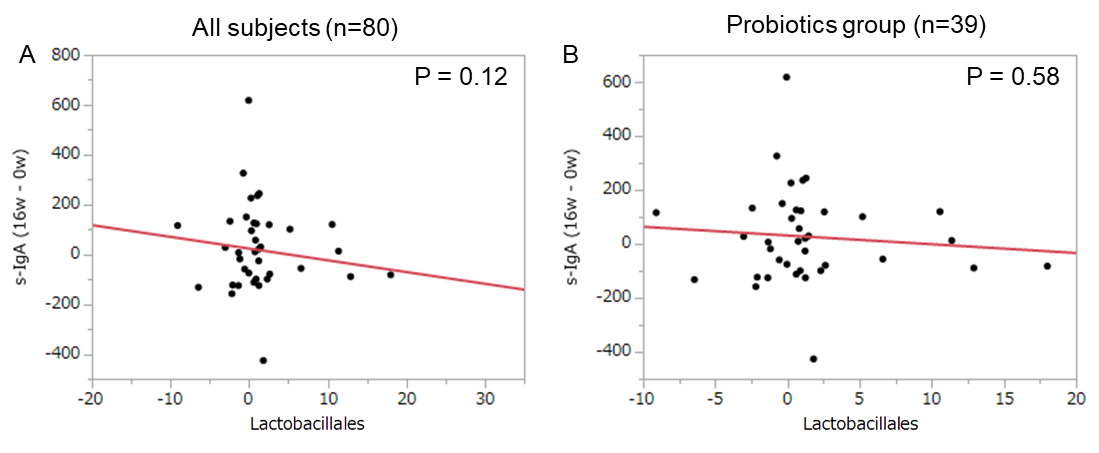


Figure S1. Correlation of occupation ratio of Lactobacillales to amount of s-IgA in saliva.

The ratio of occupation and amount of s-IgA were relative to baseline 16 weeks after start of trial. P values were calculated by linear regression analysis.
