# Supplementary material for: Leuconostoc mesenteroides subsp. strain NTM048 ameliorated nasal symptoms in patients with Japan cedar pollinosis: Randomized, double-blind, and placebo-controlled trial
Source: Medicine (Baltimore). 2023 Nov 10;102(45):e35343. doi: 10.1097/MD.0000000000035343 (PMC10637569; doi:10.1097/MD.0000000000035343)
Supplement: Supplementary file 4 [file medi-102-e35343-s004.docx]

Figure S2.


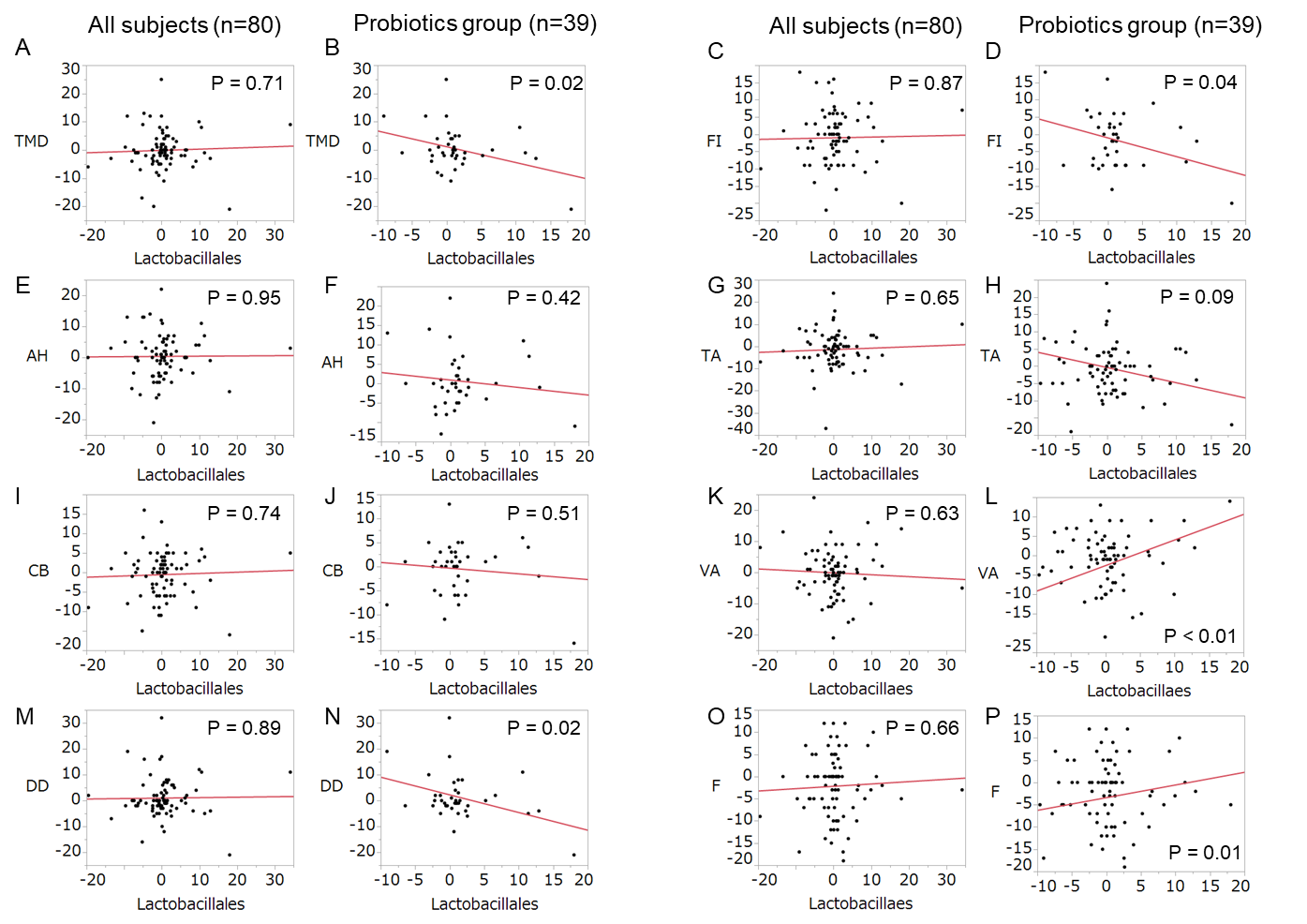


Figure S2. Correlation between t-scores in POMS2 and the occupation ratio of Lactobacillales. P values were calculated by linear regression analysis.
