# Supplementary material for: Leuconostoc mesenteroides subsp. strain NTM048 ameliorated nasal symptoms in patients with Japan cedar pollinosis: Randomized, double-blind, and placebo-controlled trial
Source: Medicine (Baltimore). 2023 Nov 10;102(45):e35343. doi: 10.1097/MD.0000000000035343 (PMC10637569; doi:10.1097/MD.0000000000035343)
Supplement: Supplementary file 5 [file medi-102-e35343-s005.docx]

Figure S3.


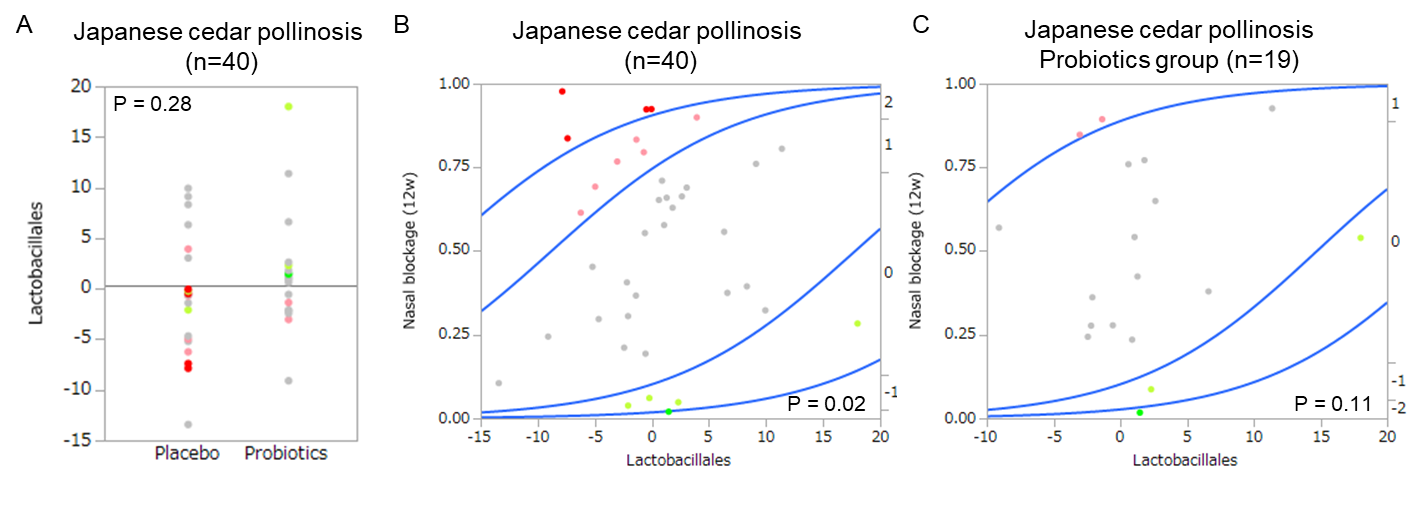


Figure S3. Analysis of correlation between nasal blockage and the occupation ratio of Lactobacillales. Red markers indicate patients with JCP whose nasal blockage was aggravated 12 weeks after start. Green markers indicate patients with JCP whose nasal blockage was improved 12 weeks after start. Thickness of markers indicate the degree of worsening or improvement. P value was calculated by Wilcoxon test (A), and other P values were calculated by logistic regression analysis (B and C).
